# The Incidence of Concussion in a Professional Australian Rugby League Team, 1998–2012

**DOI:** 10.1155/2013/304576

**Published:** 2013-12-03

**Authors:** Jason Savage, Chloe Hooke, John Orchard, Richard Parkinson

**Affiliations:** ^1^Monash Health, Department of Oral and Maxillofacial Surgery, Melbourne, VIC 3168, Australia; ^2^Monash Health, Melbourne, VIC 3168, Australia; ^3^School of Public Health, University of Sydney, Sydney, NSW 2006, Australia; ^4^St Vincent's Hospital, Department of Neurosurgery, Sydney, NSW 2010, Australia

## Abstract

*Background*. Rugby league is a physically demanding team sport and the National Rugby League is the highest-level competition of rugby league in Australia. Frequent tackles and collisions between players result in a high incidence of injury to players. Concussion injuries have been the source of much debate, with reporting varying greatly depending on the definition used. *Method*. Injury records of 239 players from one professional National Rugby League were analysed during a continuous period of 15 years, with particular interest in the incidence and recurrence of concussions and the change in incidence over time. *Result*. A total of 191 concussions were recorded, affecting 90 players. The incidence of concussion injuries was found to be 28.33 per 1000 player match hours, with an increase over time (*P* = 0.0217). Multiple concussions were recorded for 51 players. *Conclusion*. A statistically significant increase in the incidence of concussion injuries was found, without a concurrent increase in the number of head injuries or total injuries. New rules which mandate removal of players from the field may be beneficial for protection of players on the long term, although they risk being counterproductive, if they make players less likely to report their symptoms during matches.

## 1. Introduction 

The National Rugby League (NRL) is the highest level of professional competition of rugby league in Australia. The playing season runs from March (autumn) to October (spring) each year. Rugby league is a full-body contact, physically demanding team sport consisting of 13 on field players: 6 forwards, 7 backs, and up to 4 interchange players [1–5]. Forwards are chosen for their size, strength, and tackling ability as they are involved in more body contact, whilst backs are often chosen for their speed and agility and are less involved in defensive physical contact [2, 3, 6, 7]. Compared to rugby union, contests for the ball in rugby league are decided in favour of the team in possession for up to 5 tackles with a handover occurring on the 6th tackle to the opposing team [1, 2, 4]. Player tackles and collisions make rugby league a popular spectator sport, but also one with a high reported incidence of injury [8]. Concussion is an injury with relatively low incidence if the injury definition requires players to miss a match, but higher incidence if “non-time-loss” injuries are taken into account [8–12].

## 2. Aims

A review of the literature regarding injuries occurring in rugby league revealed that previous studies have been of a short duration, and are not concussion injury specific or used a definition of injury that relied on the affected player being unavailable for the following match likely under reporting the true incidence of concussion injuries [8, 13, 14]. This study was conducted at a professional rugby league team where one of the authors (JO) has kept continuous injury records of injuries managed by the team medical officer since the inaugural season of the NRL, in 1998. The purpose of this study was to identify all presenting concussion injuries and their severity, during the 15-year recorded period for this professional rugby league team. Our aim was to describe the true concussion incidence and consider any changes over time. We hope this study will promote further research into the monitoring of concussion injuries and aid in awareness of the risks of multiple concussion injuries.

## 3. Method

Injury records for a cohort of professional rugby league players competing in the NRL, from 1998 until the conclusion of the 2012 season, were made available for retrospective analysis. During this period all injuries that occurred were recorded by the team's medical officer. This sports physician was employed for the duration of the study and was present at >95% of matches. Injuries were recorded in accordance with protocols described by Orchard using a modified Orchard sports injury Classification System [15].

Data was collected and managed in accordance with the Declaration of Helsinki for the Conduct of Clinical Research and Australian National Health and Medical Research Council guidelines [16, 17].

Injury data included details of the following:

- nature of the injury,
- site of the injury,
- time of injury onset,
- team played for and against,
- time loss as a result of injury.

In the setting of multiple player injuries, all injuries were recorded.

An injury was defined in this study as “any physical or medical condition that required a player to receive medical attention” [18, 19].

In the context of this study, a concussion was defined as any neurological disturbance reported by the player to the team doctor, no matter how transient (and irrespective of whether the player had fully recovered by the time he was examined) [20–22]. A recurrent concussion injury was defined as “any subsequent concussion injury that occurred after a player had returned to full team participation from the index concussion,” without necessarily being in the same season [23]. The definition of a concussion for this study differs from some recent interpretations of concussion guidelines, in which a borderline condition is recognised (i.e., transient headache ± dizziness which quickly resolves but does not involve unconsciousness, amnesia, or prolonged symptoms) [24, 25]. It is not clear whether these “mild” cases are necessarily concussions and whether they should be removed from the field, but for the purposes of this study they have been recorded [25, 26].

During the study, the number of players on the field remained constant at 13 per match, with 4 players on the interchange bench. All participants were male, aged 18 and over.

Statistical analysis consisted of the calculation of the injury incidence. The injury incidence is reported as the number of injuries per 1000 player match hours (1000 pmh), where

Incidence = 1000 ∗ (number of injuries per season)/1.33 (80 minute matches) ∗ (number of matches per season) ∗ 13 (on field players) [27].

A regression analysis of the incidence of concussion injury, head injury, and total number of injuries was performed using the R software package and the data was fitted using a linear model with hypothesis testing for a nonzero slope.

Additionally the incidence of injury between the playing positions of forward or back was compared as a difference between 2 proportions (of those playing hours where there was an injury).

## 4. Results

**Figure 1:**
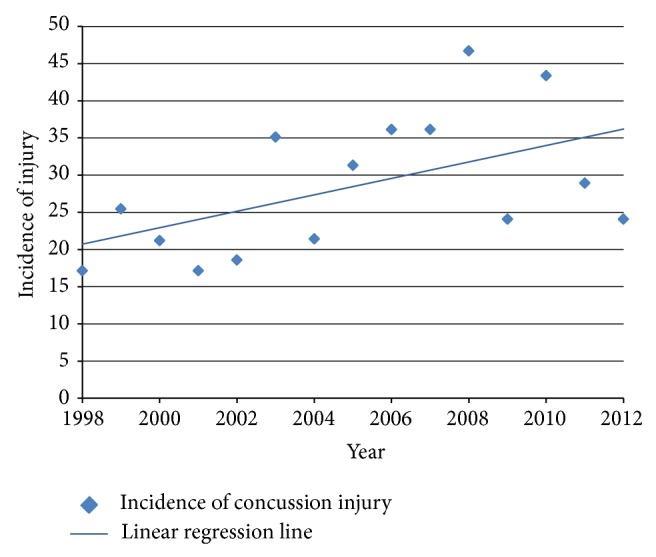
Incidence of concussion injuries. (Slope of regression line of the incidence of concussions = 1.11).

The cohort consisted of 239 players, 99 that played a single NRL season and 140 that played multiple NRL seasons. During 15 consecutive professional NRL seasons a total of 191 concussions were recorded, affecting 90 players. 101 concussions were recurrent injuries according to our study definition (i.e., in the setting of a recorded previous concussion at this team at any time in the past). The exposure time of the study was calculated at 6743.1 hours of professional NRL competition, over 390 matches. The incidence of concussion injuries was calculated to be 28.33 per 1000 pmh. We found that the concussion incidence increased over time, with the linear regression slope found to be 1.11 ± 1.07 with 95% CI (hypothesis test passed: *P* = 0.0217) (Figure 1). The incidence of repeated concussion injuries was found to be 15.13 per 1000 pmh during the 15 consecutive seasons.

The total number of head injuries (excluding concussion) sustained during matches in the study period was 386. The total number of injuries sustained to players during the study period was 2352 (Table 1). The number of head injuries and the total number of injuries remained fairly constant over the study period, with no statistically significant increase in later seasons (head injury *P* = 0.349, total number of injuries *P* = 0.319).

Multiple concussions during the course of the study affected 51 players. Up to 7 concussions to the same player were recorded. Within the same season 27 players sustained a second concussion, 4 players sustained a third, and 2 players sustained a fourth (Table 2).

Players in a forward field position recorded a higher incidence of injury than those in a back, with an incidence of 32.1 compared with 25.1 injuries per 1000 pmh (Table 3). There are 6 forwards and 7 backs on the field. One would expect that as all positions on the field have equal exposure the result should be comparable. This was found to be clinically but not statistically significant (*P* = 0.081). The position of hooker sustained the highest incidence and recurrence of concussion injuries.

Concussion injuries caused a total of only 7 matches to be missed, with 2 matches missed by players due to multiple injuries including concussion. All other players who sustained a concussion were eligible to play the following match. In only one case did a player sustain a subsequent concussion in the following match, which constitutes a recurrence rate of <1% if only the return to play match is considered. Fitness to play in subsequent matches was allowed if

1. the player no longer reported any symptoms of concussion,
2. scores on neuropsychological testing had returned to baseline levels or above. Neuropsychological testing in the first ten years was performed with a DSST (Digit Symbol Substitution Test) and over the last five years with Cogsport, a reliable cognitive function test [28].

During the study period, no associated structural brain injury and fatal or nonfatal catastrophic injuries were reported. Although it was not specifically measured as part of the study, no player was forced into compulsory medical retirement from rugby league due to sequelae of concussion. However, the author who was the team doctor (JO) can recall 3 players who retired at a relatively young age (mid-20s) who cited multiple reasons for choosing early retirement. All three players sustained multiple concussions in a single season, not necessarily in their last season, and all three players were forwards. Two of the three players also played NRL at previous clubs. On the players request, a player can withhold the medical records at the time of club transfer. This can lead to an underestimation in the number of injuries the player may have sustained.

## 5. Discussion

This study found that 191 concussions occurred during the 15 seasons of the study. A statistically significant increase was found in the reported concussion incidence. A low of 17.1 concussions per 1000 pmh was found in 1998 and 2001, and a high of 46.7 concussions per 1000 pmh was found in 2008. In 2 of the last 5 years of the study the incidence rate was over 40 concussion injuries per 1000 pmh, whereas in the previous 10 years of the study, concussion injuries had never exceeded 36. There has been a statistically significant increase in concussion injuries, without a significant increase in the number of head injuries (excluding concussions), such as contusions, lacerations, facial and dentoalveolar injuries, or total injuries over the same period. A similar finding was made in amateur Rugby Union; Swain et al. found a significant increase in concussion incidence without an increase in head injuries [27].

The statistically significant increase in rate of reported concussions could be due to

1. the increased degree of reporting to the club doctor. Over the years the “team doctor” position has gradually increased from a consultant position (attending games only and then otherwise seeing players at clinic) to a permanent part-time role (including regular clinics/sessions being conducted at the club training ground). Greater contact of the doctor with players may have increased the likelihood that players will report symptoms to the doctor. Greater player education about the potential long-term effects of concussion may have also increased likelihood of reporting symptoms to the doctor,
2. a true increase in the actual rate of concussion,
3. an increase in professionalism of clubs and athletes,
4. the differences in the reporting of concussion, due to differences in definition and assessment.

The concussion incidence observed in this study (28.33 per 1000 pmh) is considerably high when compared to the incidence described by other studies. In professional Rugby League, the incidence of concussion has been reported as 8 to 8.1 per 1000 pmh [13]. In a professional AFL team, another prominent male contact sport in Australia, the incidence of concussion over a 10-year period was reported to be 16.7 per 1000 pmh when considering all presentations to medical staff [29], which is higher than rates reported when only injuries causing missed games are reported.

We found that 90 of the players monitored sustained a concussion, yet 191 concussions were recorded, 101 of these concussions were recurrent. Multiple concussions were sustained by 51 players, the majority sustaining a second (22) or third (17) concussion, with 6 a fourth, 4 a fifth, 2 a sixth, and 1 a seventh over the study. The average duration of time between the first and second and second and third concussions gradually reduced from 583 days to 309 days. Due to the small numbers of players who sustain a fourth or subsequent concussion, it is difficult to draw conclusions about the decreasing number of days between these concussion injuries.

Depending on the definition of recurrence chosen, concussion could be seen as an injury with a very low recurrence rate (<1%) the following week (if formal neuropsych testing is used to clear return to play) but a much higher recurrence rate over the course of a player's career (e.g., >50%). This is similar to another football code where in depth analysis has been conducted [30]. These divergent rates of recurrence are relevant to the two separate questions which are considered after every concussion—(1) is it safe for this player to return to play this week? (low recurrence rate if cleared by neuropsych tests) compared to (2) is it safe for this player's long-term health to continue playing a contact sport? (high recurrence rate considering career incidence).

The concussion incidence of the forwards was 32.1 per 1000 pmh compared with 25.1 per 100 pmh for backs;however, this was found not to be statistically significant. Findings were consistent with other studies that found that the incidence of injury was higher for forwards than for backs [7, 9, 31]. A contributing factor may be that certain positions on field attract players with specific physical characteristics, which may increase their risk of injury [3].

We found that the forward position of hooker was the player most at risk of sustaining a concussion injury, with an incidence of 48.2 per 1000 pmh. This was 4 times more likely than a player in the lock position, which sustained the lowest incidence of injury, with an incidence of 11.6 per 1000 pmh. Hookers have a heavy offensive and defensive role; they are positioned in the middle of the defensive ruck and are responsible for restarting offensive play, distributing the ball to support players after each “play the ball” stoppage [7]. Meir found that the hooker is the second lightest player on the field, while Gabbett found that they are the lightest and shortest of the 13 on-field player positions [3, 6]. The hooker has been found to have a significantly higher risk of injury from falling and stumbling injuries [7]. This may explain why the hooker has an increased risk of concussions, as they are constantly exposed to physical contact at head height by larger, heavier opponents. Gabbett believed that the hookers significant involvement in critical phases of play, both offensive and defensive, would result in a greater risk of injury to the player [7]. To rectify this, he suggested in 2005 that hookers could benefit from position-specific injury prevention programs, such as agility training to reduce the number of injuries from falling and stumbling [7].

Multiple concussions were sustained in the same season by 33 players. Of these players, 27 player sustained a second concussion, 4 players sustained a third concussion, and 2 players sustained four concussions within the same season. This finding was similar to what Gibbs had described, a high rate of concussion recurrence in AFL players who had sustained a previous concussion [29]. In his study over 10 years, 20 AFL players had a second concussion and 3 players a third concussion within the same season [29].

Recent changes made by the NRL will mean that “any player suspected of having a concussion must be removed from the game and be assessed by the first aider” and “a player who has suffered a concussion must not be allowed to return to play in the same game” [32]. However, clarification of this rule has indicated that it relates to clear-cut cases of concussion (e.g., unconscious, unsteady, or amnesic player) rather than the borderline symptoms (e.g., transient headache and dizziness which quickly resolves). The AFL advises that in the management of concussion injuries every case should be assessed by a medical doctor and that “if in doubt sit them out” as approach should be taken [33]. That is, “in general, the safest course of action is that the player not be allowed to return to play in the match or training session” [33].

This approach is supported by the “International Conference on Concussions in Sport,” which suggests that a player diagnosed with a concussion should not be allowed to return to play in the current game [20, 21]. Similarly in the United States since May 2009, the Zachery Lystedt Law has legislated that youth athletes “shall be removed from competition” and “may not return to play until the athlete is evaluated by a licensed health care provider” [34]. Although these policies act to make sports safer by protecting concussed players from further injury, it has been suggested that these guidelines may lead to a change in the way that sports injuries are diagnosed on the pitch or sideline. There is concern that club doctors, whilst attempting to put player safety first, may feel pressure from the clubs that employ them to return injured players to the field as quickly as possible. The renaming of a “mild transient concussion” to “traumatic migraine” or delaying assessment of the player until the match is complete may assist this cause, meaning that a player could remain on the field for the match. Gibbs' study found that of AFL players who sustained a concussion injury only 26% were removed from the field and did not return to play in that match [29].

A concern with the more conservative concussion rule is that players may be less likely to report symptoms to the team medical staff (for an injury with no observed mechanism of head trauma from the sideline), if they personally feel safe to play and they expect to be taken off the field for reporting symptoms. In the case of professional athletes, there will always be an expectation to return to play as soon as possible [35]. We need to ensure that a return to play is not detrimental to the long-term health of the players.

The management of concussion injuries is guided by clinical assessment. Gibbs suggests that clinical assessment of signs and subjective symptoms is still the gold standard to assess if a player should return to play [29]. Computerised testing of cognitive function in players is now used as a reliable baseline and as an ongoing monitoring tool following a concussion, until symptoms are fully resolved [36].

The long-term risk to sports players of sustaining multiple concussions over their careers has been a well-publicised concern. Gronwall's study concluded that the effect of concussions has been shown to be cumulative [37]. In this context, consideration of the trends of concussion incidence and recurrent concussion frequency are immensely important. Further research into possible rule changes or new protective equipment is needed to identify opportunities to decrease the risk of concussions that face professional rugby league players.

Concussion as an injury is thought to be underreported as it often does not result in a missed game and is sometimes not even detected by the treating doctor [29]. Underestimation is expected to be a common error in studies of concussion. Within this study, there are several limitations to consider. For example, each player's first concussion during the study period was labelled as an “index”; however, this may in fact have been a recurrent concussion injury, where the true “index” concussion was sustained as a junior player, whilst playing for another club or prior to the study commencing. This may cause a skew in the data, where there are more “index” concussions in the early years of the study and more “recurrent” concussions later on in the study period. Also to be considered, the number of matches recorded within each season ranged from 24 to 30. Another source of variation was the squad size, which differed each season. This may be due to a change in the player substitution rule, where in 2001 the number of interchanges was limited to 12, from a pool of 4 players on the bench. However, since the calculated incidences in this paper are recorded in “1000 player match hours” these variations in number of matches and squad size should not cause error.

A significant limitation of this study was that the mechanism of injury was not recorded. It would have been of significant interest to identify the way in which concussions occurred in matches, particularly when considering ways to decrease the risk to players. There is also a risk of bias as the concussions were reported by an employee of the club. Baseline player neurological testing was not used over the entire study period and the diagnosis of a concussion was based on clinical judgement. However, by having just one person diagnosing the concussion injuries, the opportunity for measurement bias to occur is minimised.

It should be remembered that this study analysed players within one National Rugby League team and that concussions were recorded only when they occurred during matches. National Rugby League players change teams regularly. The majority has played ten or more years of junior rugby league and may play in other leagues such as state or national squads where they are also exposed to concussion injuries. The incidence of concussions reported to the doctor in National Rugby League matches has been recorded accurately in this study, but the true incidence of recurrent concussions is likely to be much higher. It would require accurate medical records to be maintained for each player at club, school, state, and national levels. We would suggest that until we have a national concussion database recording all concussion injuries (which exists in New Zealand), it will be difficult to fully identify the real risk that repeat concussions have on a player's life.

## 6. Conclusion

This study shows that there has been a statistically significant increase in the incidence of concussion injuries over 15 seasons in a professional rugby league team. Of note, there has not been a concurrent increase in the total number of injuries sustained to players. Multiple concussions, especially within the same season, are a cause for great concern, yet this study found that no recurrent concussions occurred within the same match and only one in the subsequent week. Whilst changes to the concussion laws of games, such as the NRL, are made in the best interests of players, these may be counterproductive, providing an incentive for players to avoid reporting symptoms. More research is required to identify the risk of repeat concussions in rugby league.

## Figures and Tables

**Table 1:** Concussion injuries and incidence.

	1998	1999	2000	2001	2002	2003	2004	2005	2006	2007	2008	2009	2010	2011	2012	Total	Average
Total concussion injuries (recurrent)	8 (0)	11 (2)	11 (7)	8 (4)	9 (5)	17 (6)	10 (9)	13 (8)	15 (10)	15 (9)	21 (12)	10 (8)	21 (11)	12 (9)	10 (2)	191 (102)	12.7
Head and neck injuries (excluding concussions)	24	18	37	30	33	29	18	22	18	31	20	23	30	29	24	386	25.7
Total number of injuries	190	142	203	138	166	125	120	143	146	163	156	149	235	167	111	2352	156.8
Number of matches played	27	25	30	27	28	28	27	24	24	24	26	24	28	24	24	390	26
Number of players monitored each season	43	41	43	46	48	49	39	38	38	41	30	27	30	30	31	239∗	—
Concussion incidence (per 1000 player hours)	17.1	25.4	21.2	17.1	18.6	35.1	21.4	31.3	36.1	36.1	46.7	24.1	43.3	28.9	24.1	—	28.3
Recurrent concussion incidence (per 1000 player hours)	0	4.6	13.5	8.6	10.3	12.4	19.3	19.3	24.1	21.6	26.7	19.3	22.7	21.7	4.8	—	15.1

^*^The total number of players monitored over 15 seasons; 140 players were monitored over more than one season.

**Table 2:** Frequency of repeat concussions and timing of injury.

Number of concussions sustained	Number of players	Portion of players injured	Average duration between concussion injuries
1	39	16.3%	—
2	22	9.2%	583 days
3	17	7.1%	309 days
4	6	2.5%	491 days
5	4	1.7%	318 days
6	1	0.5%	587 days
7	1	0.5%	204 days
Total number of players with multiple concussions	**51**	**21.3%**	**381 days**

**Table 3:** Player position concussion incidence.

Player position (number of positions)	Total number of concussion injuries (recurrent injuries)	Incidence of concussion injuries (recurrent injuries)
**Forwards**	100 (50)	32.1 (16.1)
Prop (2)	25 (10)	24.1 (9.6)
Hooker	25 (16)	48.2 (30.8)
Second row (2)	44 (23)	42.4 (22.2)
Lock	6 (1)	11.6 (1.9)

**Backs **	91 (35)	25.1 (9.6)
Halfback	14 (8)	27.0 (15.4)
Five-eighth	10 (3)	19.3 (5.8)
Centre (2)	30 (15)	28.9 (14.5)
Wing (2)	14 (3)	13.5 (2.9)
Fullback	23 (6)	44.3 (11.6)
